# Supplementary material for: The Expression Pattern of Bcl-2 and Bax in the Tumor and Stromal Cells in Colorectal Carcinoma
Source: Medicina (Kaunas). 2022 Aug 21;58(8):1135. doi: 10.3390/medicina58081135 (PMC9416041; doi:10.3390/medicina58081135)
Supplement: Supplementary file 1 [file medicina-58-01135-s001.zip › medicina-1836051-SI.pdf]

Supplementary Materials: The Expression Pattern of Bcl-2 and Bax in the Tumor and Stromal Cells in Colorectal Carcinoma

PHD	SEX	AGE	MSI	SIDE	GRADE	K-RAS	N-RAS	BRAF
1974/21	M	43	H	D	H	NO	NO	NO
3280/19	M	75	S	D	H	NO	NO	NO
3126/19	F	74	H	D	H	NO	NO	NO
2111/20	M	52	S	D	H	YES	NO	NO
55/20	F	49	S	D	H	NO	NO	YES
1785/20	M	83	H	D	H	NO	NO	YES
2251/21	M	78	H	D	L	NO	NO	NO
2727/21	F	66	S	D	L	NO	NO	YES
682/21	M	57	H	L	L	NO	NO	NO
1196/21	F	65	H	D	L	YES	NO	NO
1109/20	F	48	S	D	H	NO	NO	YES
3944/20	M	62	H	D	L	NO	NO	NO
35/21	F	73	H	D	L	NO	NO	NO
1689/19	M	57	H	D	L	NO	YES	NO
4744/19	F	81	H	D	H	NO	NO	NO
2264/20	F	34	H	D	H	NO	NO	NO
1245/21	M	39	H	L	L	YES	NO	NO
2485/21	F	67	H	D	L	YES	NO	NO
7567/20	F	69	H	D	L	NO	NO	YES
9964/18	M	73	S	L	H	NO	NO	NO
9406/20	M	83	S	D	H	NO	NO	NO
10957/21	F	54	H	D	L	NO	NO	NO
2522/20	M	51	H	D	H	NO	NO	NO
17086/19	F	74	H	D	H	NO	NO	NO
12661/18	F	65	H	D	H	NO	NO	NO
21075/19	M	58	H	D	L	YES	NO	NO
6776/18	M	72	H	L	H	NO	NO	NO
15502/19	F	80	H	D	H	YES	NO	NO
6930/21	F	77	S	D	H	NO	NO	YES
20210/21	F	63	H	D	H	NO	NO	NO
15796/20	M	71	H	D	H	NO	NO	NO
246/20	M	55	S	D	L	NO	NO	NO
12269/21	F	43	S	L	L	YES	NO	NO
2149/16	F	82	S	L	L	NO	YES	NO
8047/21	F	76	S	R	L	NO	NO	NO
13782/20	M	82	S	R	H	YES	NO	NO
11843/21	M	68	S	L	L	NO	NO	NO
118/21	F	62	S	R	L	NO	NO	NO
10096/20	F	59	S	D	L	NO	NO	YES
